# Major abdominal wall defects in the low- and middle-income setting: current status and priorities

**DOI:** 10.1007/s00383-020-04638-8

**Published:** 2020-03-21

**Authors:** Lofty-John Chukwuemeka Anyanwu, Niyi Ade-Ajayi, Udo Rolle

**Affiliations:** 1 Paediatric Surgery Unit, Department of Surgery, Aminu Kano Teaching Hospital and Bayero University, Kano, Nigeria; 2 Department of Paediatric Surgery, King’s College Hospital, London, UK; 3 Department of Paediatric Surgery and Paediatric Urology, University Hospital Frankfurt/M., Theodor-Stern-Kai 7, 60598 Frankfurt, Germany

**Keywords:** Abdominal wall defects, Gastroschisis, Omphalocele, Low- and middle-income countries, High-income countries

## Abstract

Major congenital abdominal wall defects (gastroschisis and omphalocele) may account for up to 21% of emergency neonatal interventions in low- and middle-income countries. In many low- and middle-income countries, the reported mortality of these malformations is 30–100%, while in high-income countries, mortality in infants with major abdominal wall reaches less than 5%. This review highlights the challenges faced in the management of newborns with major congenital abdominal wall defects in the resource-limited setting. Current high-income country best practice is assessed and opportunities for appropriate priority setting and collaborations to improve outcomes are discussed.

## Introduction

Congenital anomalies account for 10% of global neonatal deaths, with children in low- and middle-income countries (LMICs) being disproportionately affected [1, 2]. Major congenital abdominal wall defects (gastroschisis and omphalocele ) may account for up to 21% of emergency neonatal interventions in the LMIC setting [3, 4]. If infants with these conditions present with associated involvement of major organ systems, their management may be complicated [2, 5].

Gastroschisis (GS) and omphalocele (particularly with ruptured sac) are associated with fluid shifts and physiological alterations that make management challenging in the resource-constrained setting. This may be exacerbated by suboptimal neonatal transportation to the appropriate health facility. In many LMICs, the reported mortality is 30–100% [6–8], while in high-income countries (HICs), mortality in infants with major abdominal wall defects is less than 5% [9, 10].

This review highlights the challenges faced in the management of newborns with major congenital abdominal wall defects in the resource-limited setting.

Current HIC best practice is assessed and opportunities for appropriate priority setting and collaborations to improve outcomes are discussed.

## Gastroschisis

### Aetiopathogenesis

GS is a full-thickness congenital abdominal wall defect typically located to the right of the umbilicus. Through this defect, small and large bowel and sometimes other intra-abdominal viscera herniate into the amniotic cavity and are bathed by amniotic fluid in utero. Historically, GS was reported to have an incidence of 1 in 4000 live births. However, recent data suggest that there have been substantial increases in incidence globally over the past two decades [11–14].

It is believed that GS results from in utero involution of the right umbilical vein, which results in necrosis and a full-thickness defect in the right paraumbilical area [11, 14, 15]. Others have suggested that early involution of the right vitelline (omphalomesenteric) artery causes a paraumbilical abdominal wall defect [14, 15]. Although the exact cause of GS is unknown, young maternal age and interactions between gene polymorphism, cigarette smoking, and illicit drug use are suggested predisposing factors [16, 19].

In a retrospective review of 39 patients managed for gastroschisis at the Aminu Kano Teaching Hospital Kano (AKTH), Nigeria (Table 1), over a 5-year period, the median maternal age was 19 years (range 15–35).

**Table 1:** Selected variables of patients with gastroschisis (AKTH Kano)

Parameter	Minimum	Maximum	Median
Age on admission in days	1	6	2
Gestational age at birth in weeks	33	40	38
Age of mother in years	15	35	19
Age of father in years	25	45	30
Weight on admission in kg	1.30	3.20	2.20
Age in days at time of first surgery	1	10	3
Age in days at time of second surgery	3	13	7.5
Age in days at full oral feeds	3	18	9.5
Age in days at time of death	1	26	3
Length of hospital stay in days	1	26	3

In GS, the determinants of intestinal damage include the duration of exposure to amniotic fluid and the size of the abdominal wall defect [12, 17]. As the pregnancy advances, pressure from the closing abdominal wall defect may result in venous and lymphatic obstruction, which is deleterious to the bowel. In addition, the inflammatory effect of the amniotic fluid on the bowel results in the development of a fibrinous peel, which thickens and mats the bowel, with resultant reduction in the lumen and motility [14, 17].

Between 10 and 20% of patients with GS have an associated anomaly, the majority of which are in the gastrointestinal tract [11]. Some of the reported associations are intestinal atresia and duplications, volvulus, and Meckel’s diverticulum [11, 15, 20]. Table 2 shows the associated anomalies seen in patients in the AKTH review of GS outcome.

**Table 2:** Associated anomalies (AKTH gastroschisis study)

Anomaly	Number (*n*)	% (*n*/39)
Intestinal atresia	1	2.6
Polydactyly	1	2.6
Talipes equinovarus	1	2.6
Bowel ischaemia/gangrene	4	10.26
Pansystolic murmur	1	2.6
Total	8	20.66

### Prenatal diagnosis and care

The earliest indicator of the presence of a foetus with gastroschisis may be elevation of maternal serum α-fetoprotein (up to 9 multiples of the mean) [11, 14, 15]. Maternal abdominal sonography at 18 weeks helps to confirm the diagnosis [14]. Once GS is confirmed, other investigations may be appropriate; these may include foetal echocardiography and amniocentesis to exclude other anomalies [14, 15, 20]. Following antenatal diagnosis, delivery should be scheduled in a centre with a team of professionals that include a paediatric surgeon a and neonatologist [14, 15].

All (100%) of the patients in the AKTH Kano review were “out-born” and none was prenatally diagnosed. Similar findings have been reported from the sub-region [6, 9], and other reports suggest that two-thirds of the deliveries in many LMICs are home births [1].

### Delivery and early postnatal care

The best method, as well as optimum timing of delivery for infants with GS, is debatable [14]. Given that neonates with gastroschisis and ruptured omphalocele have exposed bowel, heat and fluid losses are a major challenge. Also, relative intestinal hypomotility makes them prone to vomiting and aspiration pneumonitis. Early management includes passage of a nasogastric (NG) tube to decompress the stomach and protect the airway, adequate fluid resuscitation, and bowel protection. In some units, the use of warm-saline soaked gauze is traditional although this in itself may promote heat loss after the warm saline cools. Nursing the neonate in a controlled thermal environment is important [11, 14, 15, 20, 21]. To prevent further damage of the bowel by kinking of the mesenteric vessels prior to surgery, the baby is nursed in the right lateral position [14, 20]. Associated anomalies should intentionally be sought for.

Given that conventional central venous catheters are not available, newborns with GS in AKTH Kano undergo cannulation of the umbilical vein with a size 5F feeding tube to provide secure central venous access. Typically, these last for about 5 days before occlusion or dislodgement. Peripheral venous access is then utilized, but, in practice, is a challenging option.

### Surgical closure of the defect

Following fluid resuscitation and stabilization of the infant with GS, return of eviscerated bowel to the abdominal cavity is undertaken. This can be carried out as a primary or staged (delayed) procedure if the bowel is viable [15, 22]. The ideal method for the reduction of eviscerated bowel is debatable; however, the condition of the herniated viscera and the level of the visceroabdominal disproportion play a role in informing the choice of technique [12, 15, 17, 21].

At AKTH Kano, primary reduction and closure is the usual choice if immediate closure will not result in excessive intraabdominal pressure or respiratory compromise [15, 17]. Some authors recommend primary closure for all babies with gastroschisis and a favourable anatomy [23], while others have shown that primary fascial closure is not practicable in up to 79% of neonates with gastroschisis [24]. In the presence of a significant visceroabdominal disproportion, delayed fascial closure is done by the placement of either a preformed or a sutured silo, with serial tucks of the silo carried out on a daily basis until the herniated bowel is completely reduced. The use of the silo closure has been shown to be associated with better physiological parameters as well as patient outcomes [25]. Another key advantage of this strategy is the conversion of an emergency clinical situation into one that can be managed in a more measured way [26, 27].

A retrospective review of the outcome of patients managed with GS in AKTH Kano between September 2011 and May 2016 was carried out; of 39 patients, 26 (66.67%) were girls and 13 (33.33%) boys, giving a female to male ratio of 2:1. Other characteristics of the study patients are displayed in Table 1. Our patients are usually admitted onto the special care baby unit (SCBU), as we do not have a neonatal intensive care unit (NICU). The patients are co-managed by neonatologists and paediatric surgeons with the surgical team assuming primary clinical responsibility. Following the initial fluid resuscitation and bowel covering, the patient is nursed in an incubator. Prior to October 2014, all patients underwent surgery under general anaesthesia (GA) in the operating theatre. Those patients, who had significant visceroabdominal disproportion and viable bowel with no intestinal atresias, had a sutured improvised silo placed under GA, using a sterile urine bag as silo material. Those patients, who had complex GS with gangrene or intestinal atresia, underwent resection of the pathologic bowel segment, a primary intestinal anastomosis, and primary abdominal wall (fascia and skin) closure (Table 3).

**Table 3:** First intervention on presentation (AKTH gastroschisis study)

Intervention	Number	%
Improvised silo bags (sutured)	11	28.2
Improvised silo bag (non-sutured)	9	23.1
Primary closure	6	15.4
Died before surgical intervention	13	33.3
Total	39	100

**Fig. 1:**
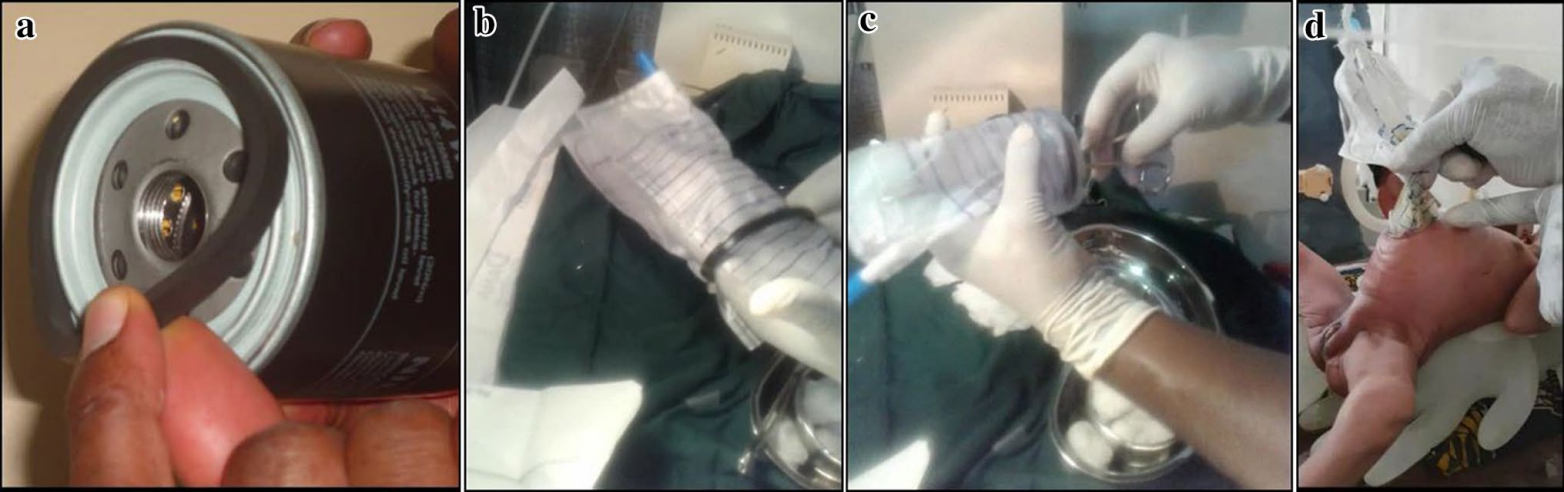
**a** Rubber ring of automobile oil filter. **b** Cut end of urine bag fitted into the rubber ring. **c** Cut end of bag everted over the rubber ring and held in place by non-absorbable sutures to form the silo bag (Kano bag)

Evidenced by the poor outcomes of patients with GS, a decision was made to de-emphasize general anaesthesia in the management of those infants without intestinal pathology, as most died peri-operatively. From October 2014, this cohort has been managed with an improvised silo placed in SCBU under sedation with IV-diazepam (0.1 mg/kg slow IV push). The silo is fashioned from a sterile urine bag and a rubber ring from an automobile oil filter (Fig. 1a–c). We sterilize the rubber ring by first washing with a detergent and soaking in activated dialdehyde solution (Cidex™) for 30 min. When this solution is not available, we soak in 10% povidone iodine for 30 min. Recently, we started to autoclave the rings and preserve them in a sterile container. All our patients received intravenous antibiotics (ceftriaxone 100 mg/kg/day and metronidazole 7.5 mg/kg/dose 8 hourly) throughout the period of their admission. None of them was ventilated.

**Fig. 2:**
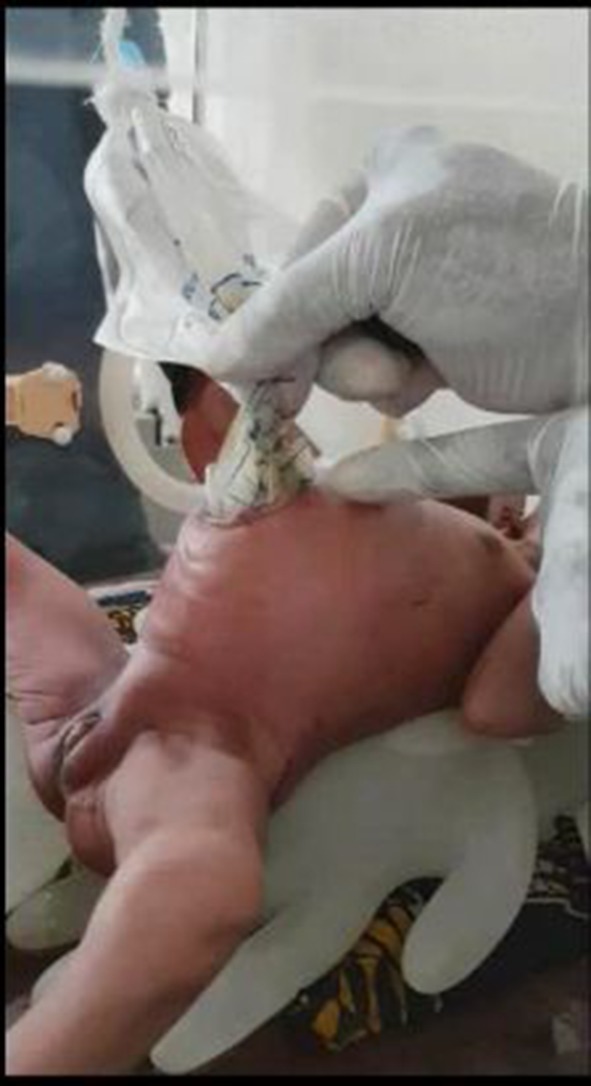
Fashioned silo bag, clinical application and complete reduction of the bowel

Following complete reduction of the bowel into the peritoneal cavity (Fig. 2), the abdominal wall is closed under local anaesthesia and sedation (muscle and fascia separate from skin) with non-absorbable sutures (Nylon 2/0) and dressed. The baby is returned to the incubator nil per os (NPO) with continuous nasogastric (NG) tube drainage and IV fluids. If by the second day post-closure, the NG-tube aspirate has reduced significantly or the baby is passing stools, the baby is commenced on breast milk that is increased as tolerated.

### Treatment outcome

Noting the high mortality rate in infants with gastroschisis who had a bowel pathology (bowel stenosis, bowel atresia, bowel ischaemia, or bowel perforation), Molik and colleagues categorized GS into two groups, i.e. simple (without intestinal pathology) and complex (with intestinal pathology), to aid the comparison of outcome between series and the categorization of risk, and this has been adopted by many [9, 21, 28–30]. Given that the causes of death in infants with GS are diverse [10, 27, 31], the capacity of a particular centre to address the various aspects of the compromised neonate with gastroschisis will impact on their outcome. Ford et al. had demonstrated that avertable DALYs (or unmet need) in the management of neonates with GS were lowest in HICs and highest in low-income countries (LICs). They concluded that GS outcomes for any particular unit are a useful barometer of the capacity of that unit to deliver generic neonatal surgical care [10].

**Fig. 3:**
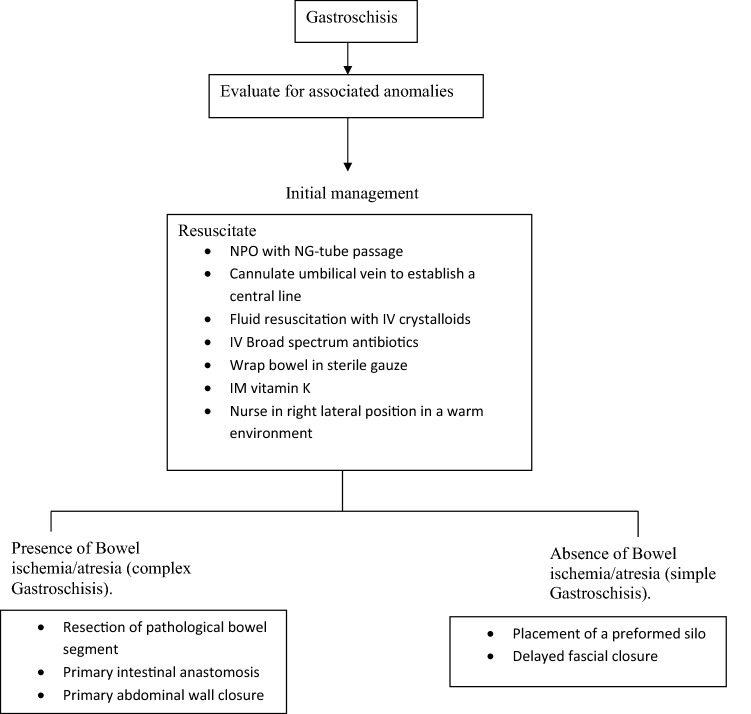
The AKTH Kano gastroschisis algorithm

Mortality in the AKTH Kano series was 87.2% (34/39). All the patients were born outside the hospital; 21 (53.8%) were septic and all were hypothermic at presentation. Earlier studies from our sub-region had shown that septicaemia and hypothermia were common causes of death in infants with gastroschisis [6, 8, 10]. Analysis of certain patient-related variables in this series, however, did not show any that was significantly associated with mortality (Table 4). Late presentations and unaccounted for system-related variables may be responsible for the recorded deaths. Figure 3 shows the treatment algorithm for the management of gastroschisis in AKTH Kano.

**Table 4:** Association between mortality and selected variables of the neonates with gastroschisis (AKTH study)

Variable	*n*	Died	Alive	Statistical significance	
				χ^2^	*p* value
Sex					
Male	13	11	2	0.115	0.735
Female	26	23	3		
Sepsis on presentation					
Yes	21	20	1	2.644	0.162
No	18	14	4		
Molik’s classification					
Simple	34	30	4	0.264	0.517
Complex	5	4	1		
Birth weight (kg)					
< 2.5	26	24	2	3.005	0.119
≥ 2.5	10	7	3		
Gestational age (weeks)					
< 37	7	7	0	0.756	1.000
≥ 37	20	18	2		
Age on admission (days)					
1	18	16	2	0.087	1.000
> 1	21	18	3

It has been shown that the provision of parenteral nutrition to the neonate with GS during the prolonged period of post-operative ileus was critical to survival [21, 32]. Total parenteral nutrition is not available in our centre. To circumvent this, when we have several patients who require parenteral nutrition at the same time, their parents take turns to buy the daily amino acid infusions which can be shared among the neonates (a 200 ml bottle costs about 14USD). We infuse this along with 10% dextrose infusions as ‘partial parenteral nutrition’. The intralipid component is not easily available. It is usually difficult for one family to sustain this for a length of time.

Prior to October 2014, we had no survivors of GS. The only survivors in this series were from the group of patients managed with the improvised sutureless silo. The consistent availability of total parenteral nutrition (TPN) may have resulted in additional survivors. Some of those who died in this group had reduction and abdominal wall closure, only to starve to death from the prolonged ileus. Analysis of our first ten cases managed with this method showed a 60% mortality rate, which was significantly different (*p* = 0.0351) from the outcome of our 11 patients managed with sutured silo under general anaesthesia.

### HIC lessons learnt and LMIC priorities for GS

Over a period of 50 years, the outcomes of GS in HICs have been transformed. Allowing for resource constraints in LMICs, lessons can be learnt and outcomes improved in considerably shorter timeframes.

The HIC improvements have come from intentional planning and multi-disciplinary working at every phase of the journey of the mother, foetus, and then baby born with GS. These include the accurate confirmation of diagnosis during pregnancy, the monitoring of the pregnancy to pick up potential complications, and planned delivery at a centre with suitable expertise to manage the baby.

Standardized antenatal scans are taken for granted in the majority of HICs, but in many LMICs they are difficult to access, provision may be poorly regulated, and reliability is variable. In a recent series of ten infants born with GS in Kampala who underwent antenatal ultrasonography, only one was diagnosed [6]. Recommendation of antenatal scans as part of the standard package of care by the WHO would signal the importance of this aspect of obstetric care and could trigger the building of the relevant capacity to deliver it and contribute to improved outcomes for infants with GS among others. Regulation of training in public and private institutions would be important to develop and maintain standards.

Where there is a high proportion of out-born infants, developing networks of referral and care are an important contributor to the survival of infants who require intervention. This means that pre-hospital care, particularly resuscitation, bowel care, and principles of newborn transportation, can be standardized by local training and the use of protocols.

Milestones in the improvement of GS outcomes in HICs included better recognition of the effects of abdominal compartment syndrome and the taking of steps to avoid and ameliorate these. These steps have included the use of improvised silos and, more recently, preformed silos to facilitate more gradual bowel reduction and the control of peritoneal fluid loss. These same technical strategies mean that reliance on general anaesthetic can be reduced, as this in itself may contribute to mortality in the sick, septic, hypothermic newborn.

While in HICs, the provision of consistent venous access is newborns with GS is given, in LMICs this can be very challenging. One of the solutions is highlighted in this report: the use of umbilical venous access to allow resuscitation, stabilization, and early nutrition. This can then be replaced in due course. Line care must be meticulous and infective complications closely monitored.

Over decades, HICs have built the capacity to deliver neonatal and paediatric intensive care. This provides the means to deal with those infants with a degree of abdominal compartment/respiratory compromise that is not available to colleagues at many LMIC settings. This situation could be improved with concerted national efforts and partnerships that will help make intensive care facilities more widely available in the LMIC setting.

The use of standardized parenteral nutrition in HICs has, unquestionably, provided the support to allow large numbers of infants to survive that hitherto would not have. However, infants in LMICs often do not survive the first few days of the condition. The data from Kampala [6] reflect death within the first 4–5 days of life in the majority of their patients. Therefore, while parenteral nutrition is a key component of modern-day care, early resuscitation and the avoidance of compartment syndrome appear to be even more important.

## Omphalocele

### Aetiopathogenesis

Omphalocele is a midline anterior body wall defect which is covered by a membrane. From inside outwards, the membrane covering the omphalocele defect consists of peritoneum, Wharton’s jelly, and amnion [11, 14, 15, 20].

Usually, the umbilicus inserts into the membranous sac of the omphalocele defect [17]. Its incidence is 1–3 per 10,000 live births, and appears to be stable so far [11, 18, 33]. The aetiology of omphalocele is not known with certainty; it is however believed to result from an abnormality of body wall infolding such that one or more of the folds (cephalic, caudal and two lateral) which close the ventral body wall at the umbilicus do not progress to the region [5, 11, 17]. Cephalic folding deficiencies result in an epigastric omphalocele commonly seen in pentalogy of Cantrell [11, 17, 34]. Lateral folding defects give rise to the commonly seen mid-abdominal omphalocele [11, 17], while deficiencies of infolding involving the caudal fold give a hypogastric omphalocele as in patients with cloacal or bladder exstrophy [5, 11, 17].

Children with omphalocele have > 50% risk of having an associated anomaly, and about 50% of these have a genetic anomaly such as trisomy 13, 14, 15, 18 and 21, and about 30–50% have a cardiac anomaly [11, 15, 33, 35]. Beckwith Wiedemann syndrome (omphalocele, macroglossia, and gigantism) is said to occur in about 10% of infants with an omphalocele [11, 35]. Infants with an omphalocele are believed to have a greater mortality risk than those with gastroschisis, because of their associated anomalies [18, 19, 33, 35]. Table 5 shows the associated overt anomalies seen in patients with omphalocele in AKTH Kano (Sept. 2011–February 2017).

**Table 5:** Associated anomalies (AKTH omphalocele study)

Anomaly	Number (*n*)	% (*n*/40)
Beckwith Wiedemann syndrome	5	12.5
Myelomeningocele	2	5
Bowel gangrene	1	2.5
Cloacal exstrophy	1	2.5
Congenital heart disease	1	2.5
Down’s syndrome	1	2.5
Parasitic twin	1	2.5
Anorectal malformation	1	2.5
Total	13	32.5

### Prenatal diagnosis and care

The use of antenatal ultrasonography and maternal serum α-fetoprotein usually detects about 80% of ventral body wall defects [11, 15, 17]. Elevation of maternal serum α-fetoprotein in omphalocele is less than that seen in gastroschisis, i.e. about four multiples of the mean [11, 15].

In HICs, routine prenatal ultrasound will be performed on the basis of structured protocols to discover major abnormalities. Prenatal ultrasound is very much sensitive for identifying omphalocele. Therefore, the prenatal detection rate of omphalocele is high, between 83 and 99%, depending on the expression of the underlying disease, i.e. 83% for isolated cases, 95% of non-isolated, and 99% of chromosomal cases [36].

Most cases of omphalocele will be discovered by prenatal ultrasound at late first or early second trimester and result in high rates of termination and/or foetal loss (stillbirths). It has been estimated that around 30–49% of omphaloceles present with chromosomal abnormalities (trisomy 13, 18, 21) and the majority (85%) of these pregnancies will be terminated [14, 37]. Furthermore, major associated anomalies in prenatally detected cases of omphalocele usually will lead to a decision of termination of pregnancy. Several reports confirm a 30–61% termination rate in prenatally diagnosed omphalocele [38].

Another interesting observation is that associated anomalies appear to be more common with minor omphaloceles (2–5 cm) than giant omphaloceles (55% vs. 36%) [39]. A prenatally confirmed diagnosis of omphalocele should ultimately lead to the intrauterine transfer to a designated tertiary centre. This is to enable the decision making with regard to termination of the pregnancy or a structured pre-, peri-, and postnatal follow-up of these cases [40].

Pre- and perinatal mortality is high in omphalocele. A large cohort study revealed that 37% of the cases had termination of pregnancy or stillbirth, an additionally 15% died within the first 15 days after delivery, and that prenatally diagnosed and non-isolated omphaloceles had a significant higher mortality than postnatally diagnosed and isolated cases [41].

Delivery of prenatally diagnosed omphalocele patients is usually at term. The mode of delivery is under debate also in HICs, but should be mainly related to obstetric considerations. In fact, delivery mode is influenced by the type/size of omphalocele in some HICs (Table 6) [39, 42].

**Table 6:** Type/size of defect, usual content, and modality of birth

Size of defect	Content of sac	Delivery
Hernia to the cord	Small bowel, omphalomesenteric duct remnants	Vaginal at term
Minor defect (2–5 cm)	Small bowel/liver/stomach	Vaginal/caesarean at term
Large defect (> 5 cm)	Liver, spleen, stomach, small bowel	Caesarean at term

Adopted from Gamba and Midrio [42], Verla et al. [39]

### Early postnatal care

Like gastroschisis patients, infants with omphalocele are prone to increased fluid and heat loss. In neonates with a ruptured omphalocele, the heat and fluid loss are similar to that for infants with gastroschisis. The initial post-delivery management also consists of airway control and passage of a nasogastric (NG) tube, fluid resuscitation, temperature regulation (nurse child in a thermoneutral environment), and careful inspection of the sac to make sure it is still intact. The sac is subsequently covered in a warm saline-soaked gauze, after the child has been carefully evaluated for any associated congenital anomalies [15, 20, 43].

**Fig. 4:**
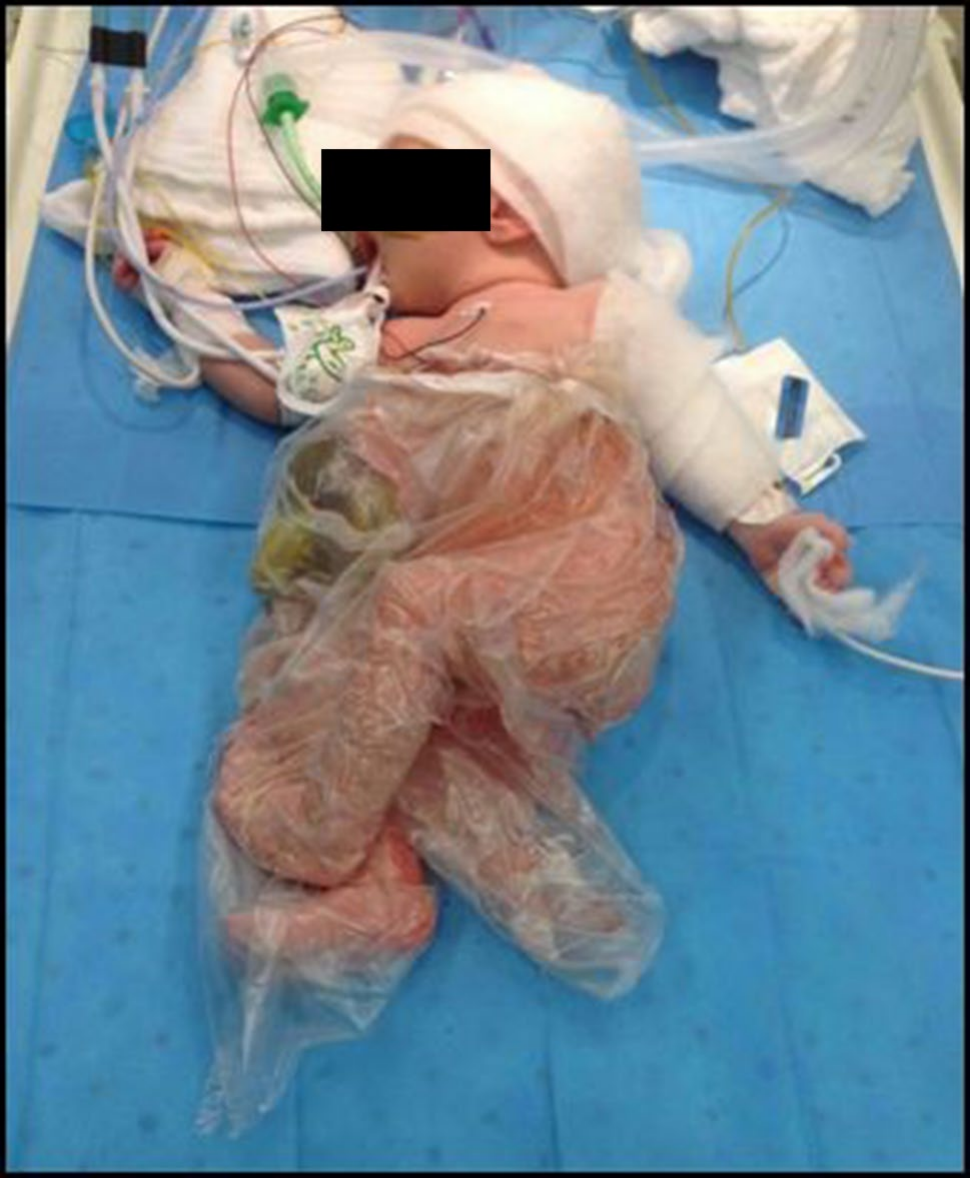
Plastic bag for primary care

Surgery of omphalocele is not an emergency procedure as long as the amniotic sac remains intact. Newborns should be placed in a sterile plastic bag (Fig. 4) to avoid fluid and temperature loss, which is less than in gastroschisis, but of course much higher when compared to newborns with an intact abdominal wall. Adequate resuscitation of the newborn and thorough assessment for additional congenital anomalies are necessary. First, the cardiopulmonary system of the newborn with omphalocele requires careful investigation, including echocardiography [44].

Concomitant pulmonary hypoplasia in giant omphalocele might require early intubation and ventilation. These cases would have been ideally already detected prenatally and would thus be known to the neonatology team. Naso- or orogastric tube is recommended for decompression of the gastrointestinal tract. An appropriate intravenous access should be secured for proper fluid resuscitation and for later surgery. If the newborn presents with a ruptured omphalocele, the treatment regime as in gastroschisis will be applied [45].

### Surgical closure of the defect

The definitive treatment of an omphalocele depends on a number of considerations such as the integrity of the sac, the size of the defect, the presence of associated anomalies, and the gestational age of the child [11, 14, 15]. As in the management of gastroschisis, the optimal approach to abdominal wall closure is debatable [11]. Depending on the size of the fascial defect, it may be classified as omphalocele major (≥ 5 cm of fascial defect) or omphalocele minor (< 5 cm of fascial defect) [15, 46, 47]. Some have used the term “giant omphalocele” to describe omphaloceles with the size of the fascial defect greater than 5 cm and which has the liver as one of the herniated viscera in the sac [48].

#### Primary closure

**Fig. 5:**
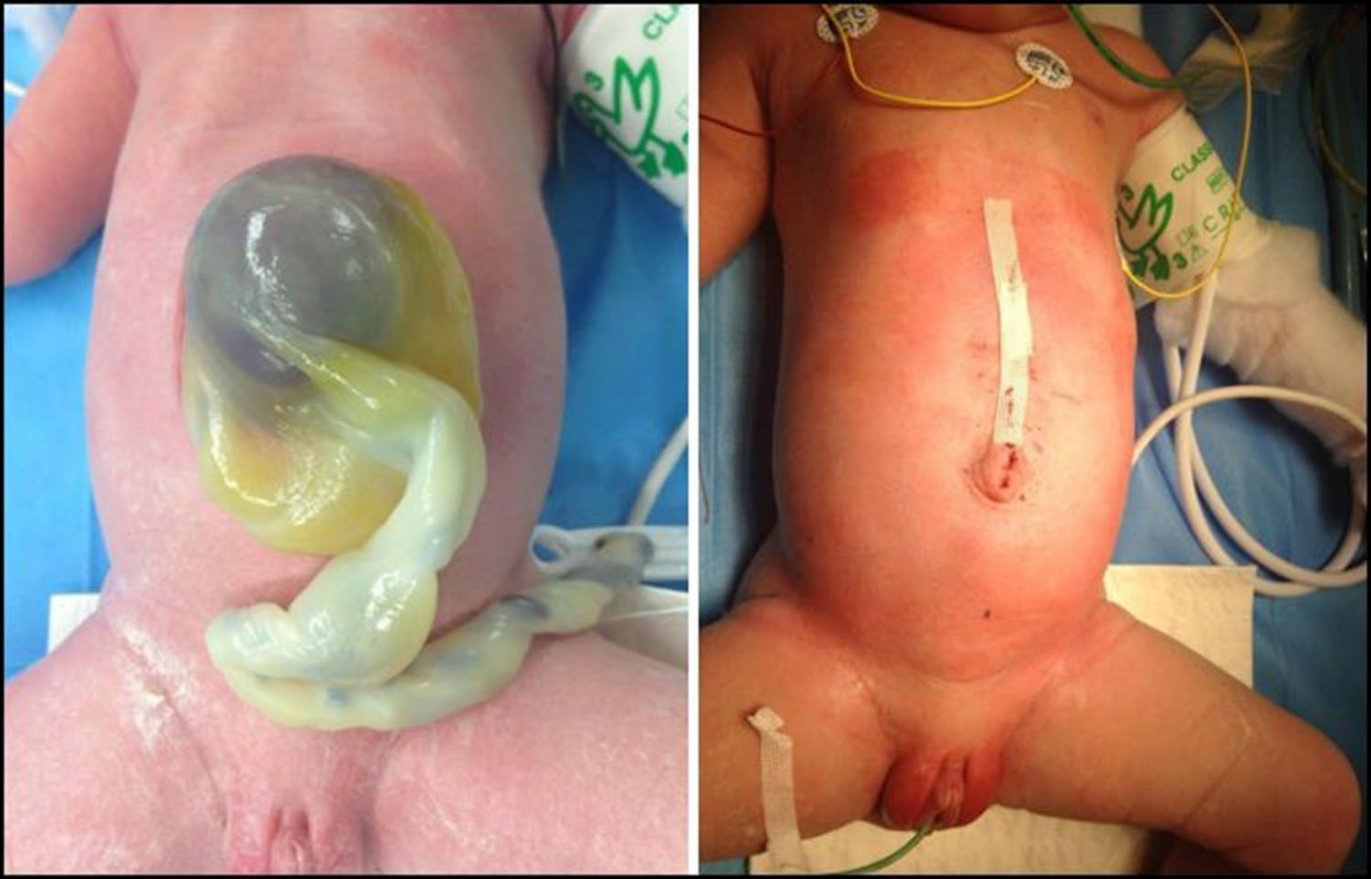
Primary closure of moderate-sized omphalocele defect

Primary closure, with excision of the amniotic sac and closure in layers (fascia and skin), is standard in small defects (2–5 cm), but could also be performed in large defects (Fig. 5).

There is no general consensus on the size of the defect and the content of the sac with regard to an attempt at primary closure. Single stage, early closure could be achieved in giant omphaloceles using either the anatomical closure in layers or the insertion of a synthetic patch into the fascia followed by skin closure [49].

##### Staged closure

Gradual reduction using the amniotic sac itself and sequential ligation would be one of the options for staged closure. Staged closure using a synthetic prosthesis constructed like a silicon chimney is a useful method in giant omphaloceles [50].

##### Delayed closure

Escharotic therapy can be done in selected cases, especially when primary closure is not feasible. Silver sulfadiazine is a commonly used escharotics agent. Patients could be kept in the ward and do not need intensive care treatment. Additionally, feeding is well tolerated during this treatment option. Treatment could be continued at home, including compressive dressing of the sac. Closure of the resulting abdominal hernia could be scheduled at the age of 6–12 months.

A systematic review of the methods of staged surgical vs. non-operative delayed closure revealed differences in mortality, length to full enteral feeding and length of hospital stay with a slight benefit seen in the delayed closure group. However, the authors did not take the necessary delayed closure surgery for the resulting ventral hernia in the non-operative closure group into account [51].

**Fig. 6:**
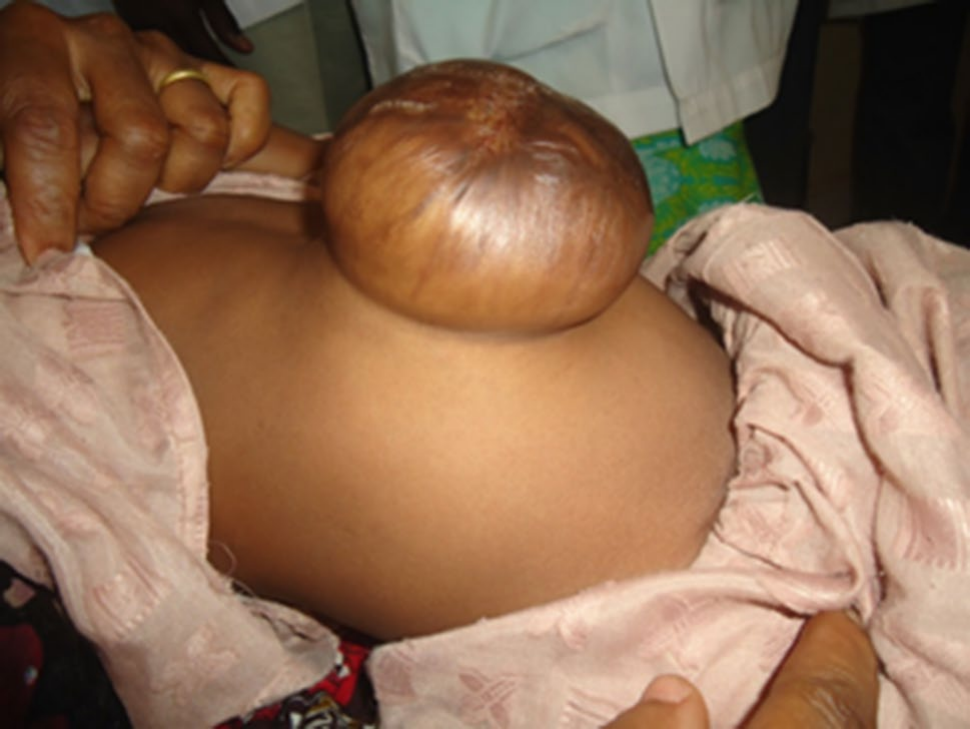
Ventral hernia formed following non-operative management of omphalocele

A retrospective analysis of patients managed for an omphalocele in AKTH Kano between September 2011 and February 2017 showed that of the 40 patients whose records were analysed, 24 were males and 16 were females (M:F = 1.5:1). Only one of the patients (2.5%) had an antenatal ultrasound scan detection of the anomaly. Of the 40 patients, 27 (67.5%) were born at home, while 13 (32.5%) were born in a health facility. There were two (5%) caesarean deliveries due to foetal distress. The majority (35/40; 87.5%) of the neonates had an omphalocele major. Six patients presented with a ruptured sac. These six patients had an operative treatment, with the placement of a sutured silo bag under general anaesthesia. All other patients with an intact sac had a non-operative treatment with the use of an escharotic agent (silver sulphadiazine cream) topically applied to the sac to induce epithelialization and create a ventral hernia (Fig. 6) which would be closed at a later date [15, 43, 47, 48, 52, 53]. Other parameters of the study patients are as depicted in Table 7.

**Table 7:** Selected patient variables (AKTH omphalocele study)

Parameter	Minimum	Maximum	Median
Age of mother in years	16	45	26
Parity (no. of births) of mother	1	10	4
Age of father in years	25	60	40
Weight on admission in kg	1.5	3.9	2.58
Length of hospital stay in days	1	47	12

#### Treatment outcome

The survival in the AKTH series of omphalocele patients was high, owning to the use of non-operative management. Similar findings have been reported from our sub-region [5, 47, 53]. The mortalities in this series were in the patients who had a ruptured omphalocele (6/40; 15%). When the sac ruptures, the infant is exposed to the same challenges as the child with a gastroschisis, with the attendant risk of body fluid and heat losses and the development of septicaemia. Given the size of the fascial defect, the improvised preformed silo cannot be used in these cases, as it cannot be held easily in place. We thus have to employ a sutured silo under general anaesthesia. Due to the size of the defect, and our inability to paralyse and ventilate the patient, reduction is slow, with the wound easily becoming infected and result in the separation of the silo from the fascia and exposing the peritoneal cavity [11]. The attendant sepsis, hypothermia and malnutrition result in a dismal outcome for such babies with a ruptured omphalocele sac [8].

The overall reported survival rate of live-born omphalocele cases is 75–81% in the current literature. The outcome for omphalocele correlates directly with the size of the defect [54].

Patients with isolated omphalocele have the best 1-year survival, which is greater than 90%, but this is a rather small group of omphalocele cases (around 30%). Giant omphalocele (defect containing more than 75% of liver in the sac and/or defect diameter larger than 5 cm) have an in-hospital mortality of up to 20% [55].

Long-term medical problems occur such as gastroesophageal reflux, pulmonary insufficiency, recurrent lung infections or asthma, feeding difficulties, and failure to thrive [55, 56]. Special attention has been focused on the neurodevelopmental outcome of patients with omphalocele. A recent study has shown neurological impairments in more than half of giant omphalocele survivors [57].

#### HIC lessons learnt and LMIC priorities for omphalocele

Omphalocele carries a higher survival rate compared to gastroschisis in LMIC. Nevertheless, thorough further research and increased awareness of this congenital anomaly would further improve the outcome. Facilitation of prenatal ultrasound would enable the early diagnosis and referral of these unborn patients to appropriate tertiary centres which will carry out further investigations and planned delivery. As in gastroschisis, out-born cases need to be resuscitated and carefully transported.

**Fig. 7:**
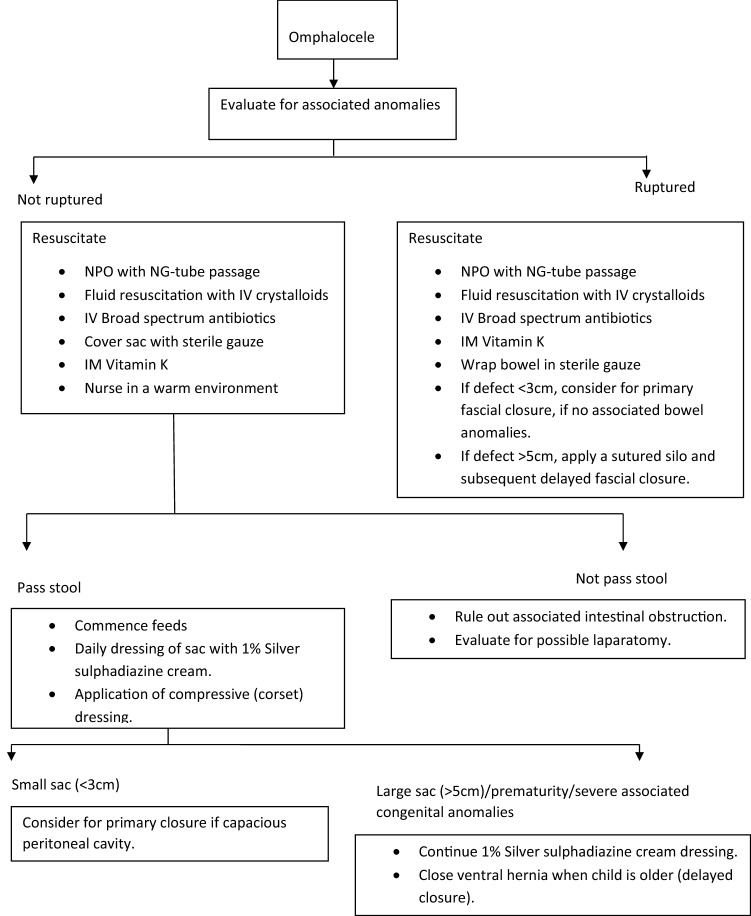
The AKTH Kano omphalocele algorithm (adapted from Sowande et al. [5])

Newborns with omphalocele require comprehensive postnatal assessment for accompanying anomalies, especially cardiac anomalies. This requires postnatal ultrasound, echocardiography, and possible X-ray (Fig. 7).

## Conclusion

The current variations in GS and omphalocele outcomes between HIC and LMIC should not be tolerated by the health-care and global community at large. Within LMICs, efforts should be directed at establishing affordable, practical bundles of care, based on the principles outlined above. In addition to rolling out service delivery solutions, clinical, implementation science, and financial implication studies should be carried out to aid impact assessments and strategic planning. It is expected that with these measures along with other generic and specific ones, substantial falls in GS and omphalocele mortality will be demonstrated soon.
